# Stress of Prematurity in the Experience of the COVID-19 Pandemic—Current State of Knowledge

**DOI:** 10.3390/life13081757

**Published:** 2023-08-16

**Authors:** Emilia Wagner, Katarzyna Bień, Aleksandra Łomża, Arkadiusz Grunwald, Żaneta Kimber-Trojnar, Aneta Libera, Bożena Leszczyńska-Gorzelak

**Affiliations:** Chair and Department of Obstetrics and Perinatology, Medical University of Lublin, 20-090 Lublin, Poland; emilia.wagner@o2.pl (E.W.); kaasiabien@gmail.com (K.B.); aleksandra.lomza98@gmail.com (A.Ł.); arek.grunwald.2@wp.pl (A.G.); anetalibera@wp.pl (A.L.); bozena.leszczynska-gorzelak@umlub.pl (B.L.-G.)

**Keywords:** COVID-19 pandemic, prematurity, stress

## Abstract

Stress is a process that triggers various physiological, hormonal and psychological mechanisms in response to a threat, which significantly affects the health of an individual. The COVID-19 pandemic introduced a lot of social changes that required constant adaptation to unfavorable conditions. The aim of the study was to assess the impact of stress related to this pandemic on pregnant women, mothers of premature infants and their families, and on obstetric complications, particularly preterm birth. A comprehensive literature review was performed using electronic databases such as Pubmed, Science Direct and Google Scholar. Keywords such as: “prematurity”; “pregnancy”; “stress”; “COVID-19” and various combinations of the above were used. Maternal stress and anxiety increase the levels of corticotropin-releasing hormone (CRH) in the placenta, which in turn affects the incidence of preterm birth and many other related maternal and neonatal complications. In addition, it was found that SARS-CoV-2 infection may increase the risk of this phenomenon. The COVID-19 pandemic has adversely affected preterm birth rates and the mental health of mothers of preterm infants, exacerbating their negative experience of having a premature baby. More research is needed to demonstrate the long-term effects of COVID-19 stress on prematurity.

## 1. Introduction

The premature birth of a child, defined as birth before 37 weeks of gestation, is becoming increasingly common worldwide. More than 50% long-term morbidity and circa 75% of perinatal mortality is caused by premature birth. Survived preterm children are at increased risk of developing neurological, respiratory and gastrointestinal complications [1].

According to the National Children’s Health Institute in Poland, every third child born before the completion of 32 weeks of gestation requires hospitalization in a neonatal intensive care unit [2].

Data published between 2015 and 2023 by the European Centre for Disease Prevention and Control (ECDC) under the Euro-Peristant project indicates that 7.1% of all live births in European countries are premature. However, the prevalence of preterm birth varies among individual countries, with Romania having a rate of 11.1% and Iceland having a rate of only 5.5% [3]. Generally in the world it varies between 5% to 18%. Despite the fact that society is constantly evolving, this rate does not decrease because new causes arise in developing countries [4].

According to the World Health Organization (WHO), approximately 14.9 million premature babies were born worldwide in 2021, accounting for around 10.6% of all births [2]. It should be noted that due to the COVID-19 pandemic, most countries experienced an increase in the number of preterm births, attributed to factors such as increased stress among pregnant women or the direct impact of coronavirus infection [2,3,5].

Moreover, lockdown during the COVID-19 pandemic could have caused modifications in environmental and behavioral life, such as reduction of physical activity, employment, social support and the most common in this group, reduction of access to healthcare—especially gynecological and obstetric [6]. Hedermann et al. research presented a slightly increased number of very premature births, with no significant differences in rates of moderate premature, term or post-term births during the COVID-19 pandemic [7].

The comprehensive literature review was performed using the electronic databases: Pubmed, Science Direct and Google Scholar. Keywords such as: “prematurity”; “pregnancy”; “stress”; “COVID-19” and various combinations of the above were used. The search was limited by 2019 year for „COVID-19” and 2010 year for the others keywords. The aim of this review paper is to summarize the current state of knowledge about how experience of COVID-19 pandemic may have affected the stress of prematurity. Due to many research, the influence of COVID-19 pandemic in the adults is currently well-known. The importance of this review paper is focusing on the impact of stress caused by pandemic in the early, prenatal and postnatal (particularly premature) stage of life. Stress may affect the pregnancy course, or obstetric complications, as well as long-term maternal and offspring outcomes [6].

## 2. Influence of COVID-19 on Pregnancy

Both the incidence of COVID-19 in pregnant women and the impact of this infection on pregnancy outcomes are still being investigated. Existing research suggests that SARS-CoV-2 infection may increase the risk of preterm birth and numerous pregnancy complications. There have also been reports of the potential placental transport of the virus and maternal-fetal transmission of the infection; however, the risk appears to be relatively low, and the number of reports confirming intrauterine transmission of the coronavirus is limited [8,9,10].

Conducted studies indicate that COVID-19 infection increases the risk of preterm birth compared to healthy women. This may be attributed to the inflammatory state resulting from the infection, which leads to the initiation of premature uterine contractions. The authors of one study conducted on a large population of pregnant women in the United States observed that COVID-19 increased the risk of preterm birth by 60% [8,9].

Coronavirus infection can adversely affect the overall health status of pregnant women, manifesting in various symptoms such as fever, cough, muscle aches, gastrointestinal disturbances (diarrhea and nausea), and respiratory difficulties. Pregnant women are also more susceptible to developing pneumonia and acute respiratory distress syndrome, which carries the risk of severe oxygen deprivation for both the mother and the fetus. Severe COVID-19 infection can also serve as an indication for the premature termination of pregnancy for the health of the mother and the fetus, thus warranting iatrogenic preterm delivery [11,12,13,14,15,16].

Furthermore, COVID-19 infection increases the risk of gestational diabetes mellitus, gestational hypertension, preeclampsia, and intrauterine fetal demise [17].

## 3. Long-Term Complications Caused by Stress on Maternal and Offspring Outcomes

Maternal psychological stress, or prenatal stress, as well as major stressful events (natural disaster, war, pandemic) during pregnancy, has been negatively associated with gestational age, weight and length of a child at birth. Furthermore maternal stress caused by psychosocial factors, such as single-parenting, food insecurity, or lack of physical activity, have been linked to lower birth weight [18].

Fetal programming is a concept described by sensation, receivement and reaction of the fetus to the intrauterine environment. Sensitive periods of fetal programming may lead to structural and functional changes in cells, tissues and organs, and therefore, long-term consequences [19]. Critical perinatal factors, involving: interactions between environment and genes, microbiota and microbiome changes, endocrine modulation, oxygen and nutrient accessibility from the placenta, duration of gestation and other environmental or genetic factors, have major influence on organogenesis and predisposition to diseases in the future [20]. The mechanisms of fetal programming in detail are currently not known, however the correlation between in utero stress and diseases such as atopic syndromes (e.g., asthma, eczema), metabolic complications, cardiovascular disorders, increased risk of infections and cancers, have been confirmed [21]. Excessive levels of glucocorticoids during pregnancy and programming of the hypothalamic-pituitary-adrenal (HPA) axis have significant impact in immune fetal development, which hypothetically may affect future offspring’s health in oncology field, increasing the risk of lymphoma, hepatic and testicular cancer [22]. The impact of fetal programming is expressed especially in the last one—testicular cancer, which is initiated from primary germ cells or gonocytes, that go through improper differentiation during embryogenesis in pregnancy. Intrauterine concentration of estrogen, which increases circa 10 times in the female and 100 times in the male fetus, may affect invalid germ cells development. Moreover unequal maternal nutrition may also induce alternations in development of fetal cells with following risk of teratogenesis in metabolic, anthropometric and behavioral functions [23]. Another study suggested that fetal programming, by decreased size of placenta in association with large birth weight (LGA) and low placenta-to-birthweight ratio, may impact to increased risk of developing Wilms tumor in the offspring [24].

In conclusion the most common two factors, which influence on fetal programming are pregnancy nutrition and stress [19]. However other adverse intrauterine events, including maternal anxiety, depression, mental or metabolic diseases have their significant impact as well. Many studies reported that maternal anxiety or stress was linked to fetal tachycardia, sudden decreases or increases in fetal heart rate (FHR) and enhancement of fetal motor activity and breathing [25]. The most significant place of fetal programming is the human placenta, as a sensory and effector organ, which detects stress signals and activates the promoter region of the corticotropin-releasing hormone (CRH), the precursor for ACTH and β-endorphin. High levels of placental CRH are associated with preterm birth due to its endocrine, autocrine, and paracrine roles and also increasing in levels of the prostaglandins and estriol. Placental CRH during pregnancy is a significant mediator through placental receptors and vasodilatator in myometrium to potentiating mechanisms of contractions and relaxations [26].

The consequences of perinatal stress, as preterm birth and low birth weight, affects also on brain development and reduction in regional brain volume, including the prefrontal cortex, the premotor cortex, the medial temporal lobe, the lateral temporal cortex and the postcentral gyrus, which are associated with a variety of cognitive functions [25]. Thomason et al. research reported poorer connectivity between superior frontal and motor regions among neonates born preterm, which suggest impaired spontaneous motor activity in this group [27].

Maternal stress mechanism, by excess exposure of glucocorticoids, may change in utero environment in association of adverse cardio-metabolic and cardio-vascular outcomes in their offspring, such as obesity and overweight, insulin resistance, diabetes, hypertension, hyperglycemia and metabolic syndrome [28]. Van den Bergh et al. discovered that prenatal exposure of maternal stress increases the risk of unpleasant outcomes for offspring, including mental health disorders, poor cognitive functioning and behavior problems [29]. Sadman et al. found that psychobiological stress markers—especially in early pregnancy, leading to disrupted emotional regulation and impaired cognitive and motor functions during early childhood and decreased brain volume in areas associated with memory and learning [25]. Several studies suggest, that poor perinatal or fetal programming may contribute to increased risk of development psychological, neurological, or psychiatric disorders, such as schizophrenia, depression, anxiety, Attention Deficit Hyperactivity Disorder (ADHD) and Autism Spectrum Disorders (ASD) [30].

Maternal stress-involved endocrine alterations may also affect maternal cognitive brain functions and behavior. Pregnancy contributes to changes in structure and function of the brain in regions involving recognition, spatial memory and stress responsivity, which persist via lifespan [31]. Moreover Štěpáníková et al. research reported that prenatal, antenatal, or postnatal maternal stress may relapse increased risk of mental disorders, like depression among the mothers [32].

Another major concern in the modern obstetrics, that affect maternal and fetal health, is the increase of pregnancy-related cancer prevalence. The most frequent malignant tumor diagnosed during pregnancy is breast cancer, although the occurrence of other types, such as hematological, skin, or ovarian cancer, have been reported. Medical multidisciplinary interventions, especially personalized and integrated treatment, may prevent iatrogenic pregnancy complications, such as impaired fetal growth or preterm birth. Nanotechnology, as the reaction in the structures on a molecular level, improves biocompatibility, pharmacokinetics, targeting of the tumor and reduces systemic toxicity or drug resistance. Nanomedicine, as a field of nanotechnology, offers the opportunity to safe and effective application of antiblastic agents during the pregnancy. However, fetal exposure on nanoformulation, by transplacental transport is still an issue of the debate in gynecological and obstetric profession. Hence, future research is necessary to development of rational, effective and safe treatment for pregnancy-associated cancers [33].

## 4. Influence of Stress on the Pregnancy Course

Stress is a common problem among pregnant women. United States data from the Centers for Disease—Control Pregnancy Risk Assessment Monitoring showed that nearly 75% of postpartum women reported at least one major stressful event in the year prior to the birth of their child [34]. It has been indicated that 1 in 5 pregnant women will have an anxiety disorder during pregnancy, while between 10 and 14% of pregnant women fulfill the criteria for a diagnosis of major depression [35]. Pregnancy itself is associated with increased levels of experienced stress for pregnant women and the occurrence of psychological distress, anxiety disorders and depression, while the pandemic of coronavirus disease 2019 (COVID-19) has become an additional external stressor intensifying experienced stress and anxiety, additionally causing sleep disturbances in pregnant women [36,37,38]. As the level of experienced stress increases, an increase in cortisol release by the adrenal cortex is observed as a result of activation of the hypothalamic-pituitary-adrenal axis (HPA axis) [35,37,38,39]. In the third trimester of pregnancy, cortisol levels increase almost threefold, but as pregnancy progresses, hypothalamic production of corticotropic hormone (CRH) decreases, so that the HPA axis response to eustress and distress is suppressed [37] In addition, the fetus is protected from high maternal plasma glucocorticosteroid levels by the activity of the placental enzyme 11β-hydroxysteroid dehydrogenase type 2 (11β-HSD2), which converts active cortisol into metabolically inactive cortisone [37,39].

**Figure 1:**
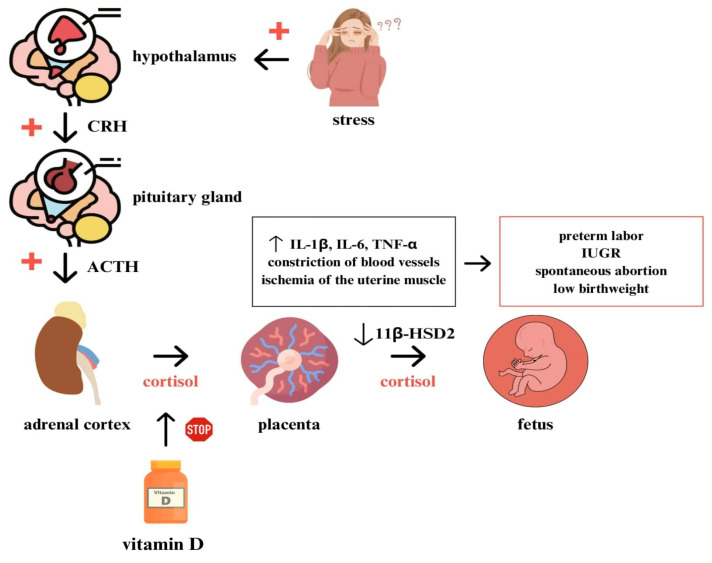
Effects of over-activation of the hypothalamic–pituitary–adrenal axis (HPA axis) on the course of pregnancy. Stress factors overactivate the HPA axis resulting in the increase of maternal corticotropin-releasing hormone (CRH) and adrenocorticotropic hormone (ACTH) levels. That results in the subsequent increase of endogenous levels of cortisol in maternal plasma and placenta, a decrease in 11β-hydroxysteroid dehydrogenase type 2 (11β-HSD2) activity and the formation of an inflammatory environment in the placenta—increased levels of interleukin-1 β (IL-1β), interleukin-6 (IL-6) and tumor necrosis factor α (TNF-α). Vitamin D reduces endogenous cortisol levels. Obstetric consequences of the described disorders include preterm labor, intrauterine growth restriction (IUGR), spontaneous abortion and low birth weight.

The HPA axis can be overactivated by external stress-inducing factors. The consequence is an excessive release of endogenous cortisol and an increase of its concentration in the placenta, the process itself is regulated by metalloproteinase-1 (MMP-1), -2 (MMP-2), -3 (MMP-3) and -9 (MMP-9) [39]. A case-control study by Duran-Chávez et al. based on 129 cases of preterm birth proved that elevated levels of MMP-9 and decreased levels of MMP-2 are positively associated with the occurrence of preterm birth [40]. There is also an increase in the release of the pro-inflammatory cytokines IL-1β, IL-6 and tumor necrosis factor α (TNF-α) suppressing the response of the maternal immune system, which may increase the risk of preterm labor [38,41]. Another mechanism responsible for this phenomenon is also the constriction of placental blood vessels under the influence of cortisol, increasing blood pressure and the formation of pro-inflammatory changes responsible for the ischemia of the uterine muscle and, consequently, the occurrence of preterm labor, as well as intrauterine fetal growth restriction [42]. Overactivity of the HPA axis is also responsible for the occurrence of depression in pregnant women and an increase in the level of already experienced stress [39]. Pregnant women diagnosed with severe depression and anxiety have higher levels of the pro-inflammatory cytokines IL-6, IL-2, IL-9 and IL-17A [35]. Elevated levels of cytokines such as IL-2, IL-6 and IL-17 are also present in the cytokine storm in the course of COVID-19 [43], one of the obstetric consequences of which is an increased risk of preterm labor [44]. Inflammatory cytokines and metalloproteinases such as MMP-1, MMP-2 and MMP-9 are responsible for the cervical ripening, at the same time, increasing the risk of preterm labor [45]. With increased levels of placental cortisol, abnormalities in placental permeability and a decrease in 11β-HSD2 activity, which is a biological marker of spontaneous abortion and preterm labor, are observed, as increased levels of MMP-9 in placental villi and tissue inhibitors of matrix metalloproteinases 2 (TIMP-2) [39]. Even a small decrease in 11β-HSD2 activity can significantly increase fetal exposure to glucocorticosteroids due to the high concentration of these in maternal plasma compared to the concentration in fetal plasma. The result is a decrease in mean birth weight [37]. Studies have shown that vitamin D supplementation may lower endogenous cortisol levels and cortisol:cortisone ratio [46]. This fact could be used to investigate how to prevent the consequences of hypercortisolemia in pregnant women. The obstetric implications of excessive activation of the HPA axis are shown in Figure 1.

Also remarkable is the fact that chronic and repeated acute stressors through modulation of the HPA axis can affect the epigenome and transcriptome, consequently causing changes related to receptors for glucocorticosteroids [35].

Maternal stress as a risk factor for preterm birth remains a major cause of maternal morbidity and mortality worldwide [38]. Beyond the increased risk of preterm delivery, prenatal stress also increases the risk of gestational diabetes and pre-eclampsia [47]. A study by Garcia-Flores et al. in a mouse model showed that intergenerational maternal stress progressively shortens the length of gestation, with the rate of preterm births in the first and second generations at 13% and 11.1%, respectively [38]. A case-control study by Lilliecreutz et al. confirmed the relationship between the occurrence of stress and shortened pregnancy duration in humans, where among pregnant women who experienced stress, 54% of them gave birth prematurely with stress as an attributed risk factor [48]. Besides stress factors directly affecting the pregnant woman, the remaining question is whether common stressors with limited impact on daily life are also a risk factor for preterm birth. An analysis by Freedman et al. aimed to show whether the assassination of John F. Kennedy influenced the occurrence of adverse pregnancy outcomes in women between 1959 and 1965. For this purpose, they analyzed data from the Collaborative Perinatal Project and showed that exposure to the mentioned factor in the first trimester of pregnancy was associated with preterm delivery (hazard ratio (HR): 1.17; 95% CI: 1.05, 1.31). Exposure in the third trimester of pregnancy was associated with an increased risk of fetal acute inflammation in the placenta (odds ratio (OR): 1.34, 95% CI: 1.05, 1.71) [49]. The effect of prenatal stress on the development of placental pathologies, which can eventually lead to spontaneous miscarriage, has also been observed. In a study by Marinescu et al. placentas of patients with high levels of social stress who experienced spontaneous miscarriage were examined, and the most common conditions included regular shape and necrotic villi, decidua with large areas of necrosis, acute inflammation and effusion areas correlated with increase in proinflammatory factors, immune deficiency and infections, hyaline type fibrosis, intervillous and deciduous intense hemorrhage [39].

Due to the adverse effects of prenatal stress on pregnancy and perinatal outcomes, it is important to identify and mitigate pregnant women’s exposure to stress early in order to prevent premature births. For this purpose, routine prenatal care should include screening for behavioral health problems and potential intervention [47]. Both untreated depressive and anxiety disorders and in utero fetal exposure to selective serotonin reuptake inhibitors (SSRIs) are associated with an increased risk of premature birth [48]. The use of benzodiazepines in combination with SSRIs is associated with an increased risk of adverse behavioral effects in infants [50]. That is why recently more and more hope has been associated with non-pharmacological methods of reducing stress during pregnancy. Methods with proven effects include meditation, biofeedback, yoga and expressive writing [35]. Data on yoga indicate that it improves quality of life by reducing stress, anxiety and sleep disturbances during pregnancy, and, in the absence of outdoor physical activity, may be effective in reducing the chance of pre-diabetes, obesity and metabolic syndrome [51]. Advantages of non-pharmacological interventions include the fact that those interventions are affordable, widely available and can all be applied without leaving home, which is particularly important in the context of the COVID-19 pandemic [35]. Used in combination with pharmacological treatment, it can be most effective for reducing perceived anxiety and stress levels in pregnant women [35].

## 5. Influence of COVID-19 on the Pregnancy Course

Both the incidence of COVID-19 in pregnant women and the impact of this infection on pregnancy outcomes are still being investigated. Existing research suggests that SARS-CoV-2 infection may increase the risk of preterm birth and numerous pregnancy complications. There have also been reports of the potential placental transport of the virus and maternal-fetal transmission of the infection; however, the risk appears to be relatively low, and the number of reports confirming intrauterine transmission of the coronavirus is limited [9,10,11,52,53,54,55]. On the other hand, the presence of the virus in the mother’s body is associated with vascular damage and complications such as hypercoagulability and deficient vascular perfusion [56]. Consequently, damage to the placenta may occur, which may facilitate vertical transmission [57]. Histologically confirmed changes in placental structure have appeared in symptomatic and asymptomatic women infected with SARS-CoV-2. To date, no adverse effects of placental damage in the course of virus infection have been observed for the child, although features of vascular malperfusion could be found in both the maternal and fetal parts of the placenta [53]. Reports of placental infection contributing to symptoms of fetal distress or stillbirth were much more common in the literature [52]. When comparing COVID-19 to other viral infections that can be acquired during pregnancy, such as CMV, Zika virus and rubella, there was also no characteristic congenital syndrome following prenatal exposure to SARS-CoV-2 [52].

Conducted studies indicate that COVID-19 infection increases the risk of preterm birth compared to women uninfected with the SARS-CoV-2 virus [58]. The authors of one study conducted on a large population of pregnant women in the United States observed that COVID-19 increased the risk of preterm birth by 60%. Taking into account the data collected from different countries, the rate of premature birth varies significantly between them and amounts to 14.3% to 61.2%. Despite the great diversity, there is a consensus that COVID-19 infection has an impact on the risk of preterm delivery. This may be attributed to the inflammatory state resulting from the infection, which leads to the initiation of premature uterine contractions. from one of the smaller studies there are reports of one type of immune cell. They express ACE2, which has the ability to cross the placenta, causing a more serious course of infection, infection of the placenta and premature birth [4,8,10]. Additionally, one study showed that women after 27 weeks of pregnancy were most often infected [59].

Coronavirus infection can adversely affect the overall health status of pregnant women, manifesting in various symptoms such as fever, cough, muscle aches, gastrointestinal disturbances (diarrhea and nausea), and respiratory difficulties. Pregnant women are also more susceptible to developing pneumonia and acute respiratory distress syndrome, which carries the risk of severe oxygen deprivation for both the mother and the fetus. Pregnant women with COVID-19 are compromised and less able to cope with the disease in general, especially when it comes to the effects of delayed diagnosis and overdue referral to treatment, when compared to non-pregnant women. The main reason is that advanced pregnancy brings about physiological and metabolic changes that have an impact on the immune system, cardiovascular function and, essential in this context, respiratory system [60]. Because the proper functioning of placenta depends on the flow of adequately oxygenated blood, the fetoplacental system, and maternal oxygen saturation during pregnancy, this virus frequently causes severe hypoxemia, which changes how oxygen is delivered to the placenta. Hypoxia and ischemia can be distinguished within the placenta through the increment in syncytial knots, while fetal hypoxia can be recognized within the circulation through the increment in erythroblasts and nuclear debris. Because the demand for oxygen rises dramatically during pregnancy, this change in blood flow and oxygenation caused by COVID-19 is extremely important because, with this compounding impact, there will inevitably be dysregulation in the oxygen supply to the placenta, resulting resulting in an increase in oxidative stress markers, which are associated with fetal health issues [61]. Meyer et al. research presented that when compared to women who were not hypoxic, women who required intubation or respiratory support due to COVID-19-related hypoxia more frequently demonstrated placental histology that showed villous trophoblast necrosis [62]. Hypoxia and ischemia accelerate fibrosis in the villous stroma [63]. The conclusion which was reached in one of the most recent reviews describing changes in the placenta of pregnant women during the three years of the pandemic is that following viral infection, maternal hypoxia can result in reduced placental blood flow and maternal vascular malperfusion, which can lead to villous infarction, hypoplasia of the distal villi, and arteriopathy in the decidua. However, confirmation of the impact of the changes described will require long-term observational programs [64].

Severe COVID-19 infection can also serve as an indication for the premature termination of pregnancy for the health of the mother and the fetus, thus warranting iatrogenic preterm delivery [12,13,14,15,16]. Van den Bergh et al. in an international cohort study discovered that maternal and neonatal complications were significantly more common in women diagnosed with COVID-19. Most notably, there was an increased number of deliveries by caesarean section and such as hypertensive disorders of pregnancy and fetal distress. Also preterm delivery rates and poorer newborn weight, length, and head circumference at birth were associated with the diagnosis of maternal viral infection [56].

Considering the mental health of pregnant women, depression is still at the first place among all mental illnesses in this population group [65]. Stress and difficulty brought on by the pandemic may cause or exacerbate typical prenatal mental health issues, such as depressive symptoms, which have been reported to have a deleterious impact on maternal-child health. In research made by King et al. they discovered that compared to women who were pregnant before the epidemic, pregnant women during the pandemic were nearly twice as likely to have probable depression [66]. Additionally, Hessami et al.’s research indicates that stress levels remained elevated during the COVID-19 pandemic while symptoms of depression and anxiety increased from the early to mid-pregnancy period [67]. During COVID-19, stress levels remained high as signs of despair and anxiety grew from the beginning to the middle of pregnancy [68]. Preliminary research suggests that increased maternal stress and depressive mood symptoms correlate with impaired fetal brain development [69].

## 6. Influence of Prematurity on the Mother and Family

The birth of a premature baby carries various consequences and implications for both the mother and the family of the preterm infant. Based on numerous scientific studies, it has been found that mothers of premature infants experience many health problems. These include infections, hypertension, increased postpartum bleeding, generalized infections, and genital tract infections. It has been demonstrated that patients who give birth before completing 37 weeks of gestation require longer hospitalization and rehabilitation [70,71,72,73].

In addition to the physical consequences of preterm birth, it has been examined that mothers who are mothers of preterm infants experience significant and enduring emotional consequences. Numerous studies have shown that mothers experience high levels of stress and anxiety, which are associated with uncertainty about the future and the health of the child. Furthermore, the separation of the mother from the child in the neonatal unit generates strong feelings of worry and fear about their development and safety. This separation alters the experience of motherhood and poses difficulties in forming a bond with the child. The necessity of making difficult medical and ethical decisions contributes to elevated levels of anxiety and stress in mothers [74,75,76,77].

Women who have experienced preterm birth are at increased risk of developing postpartum depression. The challenging experiences associated with traumatic preterm birth, separation from the child after birth, and the infant’s stay in the neonatal intensive care unit are factors that significantly elevate the levels of perceived stress, sadness, discouragement, and helplessness. It has been observed that mothers of preterm infants often blame themselves for the premature birth. They frequently experience negative thoughts and question whether the preterm birth could have been avoided or if something could have been done differently to prevent it. They also ask why it happened to them and their child. Feelings of guilt and uncertainty hinder the emotional adjustment process for women in the situation of preterm birth. It has been proven that preterm birth increases the risk of anxiety disorders and heightens the level of anxiety experienced by mothers. The stress associated with caring for a preterm child and the experience of anxiety are more prevalent in mothers of preterm infants compared to women who gave birth at term [74,76,77,78,79].

Another factor that disrupts the emotional stability of mothers is social isolation, which results from the time spent in the hospital with their child. Devoting oneself entirely to the care of the preterm infant separates and limits the mother’s contact with family and the community.

The issue of prematurity also affects other members of the family. It brings about changes in roles and responsibilities within the family. Siblings of preterm infants require increased attention and support during this time when parents focus on caring for the preterm infant. The issue of prematurity also disrupts the relationship and closeness between the father and the mother of the preterm newborn. This is a consequence of mutual blame for the preterm birth, as well as the disruption of the mother’s experience of sexuality due to the trauma of preterm birth. The family also experiences financial burdens associated with long-term medical care, including transportation to the hospital where the preterm infant is receiving care, treatment, hospitalization, and rehabilitation, which require significant financial resources. Around-the-clock care for the preterm infant, hospital visits, uncertain prognosis, and frequent stressful situations can lead to significant emotional exhaustion in the family. This has a negative impact on relationships and the well-being of the family [73,74,77,78,80,81,82,83].

## 7. The Impact of Vitamin D Deficiency Induced by the COVID-19 Pandemic on the Risk of Preterm Birth

The COVID-19 pandemic has increased the risk of vitamin D deficiency among pregnant women due to reduced sunlight exposure, particularly during periods of isolation and quarantine. This article examines the association between vitamin D deficiency and the heightened risk of preterm birth. Although the link has been studied, the underlying mechanisms remain the subject of ongoing scientific research. Potential factors that closely correlate with vitamin D deficiency and may contribute to preterm birth include the impact on the immune system, hormonal regulation, inflammatory state, and vascular function. This paper also highlights recent scientific studies conducted in different regions that explore the relationship between vitamin D levels during pregnancy and the severity of COVID-19, as well as the risk of preterm birth.

Vitamin D is an essential nutrient that has profound implications for human health, influencing various physiological processes, especially during pregnancy and reproductive health. However, the COVID-19 pandemic has raised concerns about vitamin D deficiency in pregnant women, mainly due to reduced exposure to sunlight as a result of isolation and quarantine measures. This deficiency has been linked to an increased risk of preterm birth, but the exact mechanisms underlying this association are yet to be fully understood and require further scientific investigation [84,85].

- Impact on the Immune System:

Vitamin D plays a pivotal role in the proper functioning and regulation of the immune system. Deficiencies in this vitamin can lead to an altered immune response, making individuals more susceptible to infections, inflammations, and conditions that heighten the risk of preterm birth. Calcitriol, the activated form of vitamin D, directly affects immune cells such as T lymphocytes, B lymphocytes, and macrophages, as these cells express vitamin D receptors on their surface. A 2021 study conducted by Turkish scientists aimed to assess the levels of vitamin D in pregnant women and its association with the severity of COVID-19. The study revealed a significant difference between vitamin D levels and the severity of COVID-19 in pregnant women, suggesting that maintaining adequate vitamin D levels may reduce the severity of inflammation caused by COVID-19 [85,86,87].

- Regulation of Hormonal System:

Vitamin D influences the production of hormones, including steroid hormones crucial for maintaining pregnancy. Insufficient vitamin D levels can disrupt hormonal processes and directly impact the uterus’s ability to sustain a pregnancy for its full term. The vitamin D pathway is intertwined with calcium and phosphorus metabolism and also influences the production and action of progesterone, a key pregnancy hormone. A study conducted in Bangladesh in May 2023 examined the impact of vitamin D deficiency on the course of pregnancy and found a potential link between low vitamin D levels and an increased risk of preterm birth [87].

- Inflammatory State:

Research has extensively investigated the association between vitamin D deficiency and inflammatory conditions within the body. In response to infections and inflammation, the body produces pro-inflammatory substances like cytokines, which trigger an immune response and increase the risk of preterm birth. Chronic inflammatory states accompanying vitamin D deficiency may interfere with fetal development and uterine health, leading to an elevated risk of preterm birth. A 2022 laboratory study conducted in Turin, Italy, on a group of 160 women, explored genetic variants related to vitamin D and their potential impact on vitamin D levels during pregnancy. The study found a significant correlation between vitamin D levels and a positive result in one of the SARS-CoV-2 tests, indicating that vitamin D deficiency may influence preterm birth [84,86].

- Impact on Vascular Function:

Vitamin D influences the proper functioning of blood vessels by affecting the production of nitric oxide and endothelin. Nitric oxide relaxes blood vessels and enhances their elasticity, while endothelin constricts blood vessels. Vitamin D deficiencies can disrupt these mechanisms, affecting the vasodilation and vasoconstriction processes in blood vessels, leading to impaired uterine function and reduced capacity to maintain pregnancy. A study conducted in Spain in 2020 explored the impact of vitamin D on preeclampsia and the risk of preterm birth. The findings suggested that higher vitamin D concentrations in pregnant women might decrease the risk of preterm birth and preeclampsia [86].

The COVID-19 pandemic has highlighted the potential impact of vitamin D deficiency on the risk of preterm birth among pregnant women. Although scientific studies have demonstrated the association between vitamin D levels and preterm birth risk, the precise underlying mechanisms necessitate further investigation. Understanding these mechanisms could aid in the development of preventive and therapeutic strategies to mitigate the risks associated with vitamin D deficiency during pregnancy, particularly during pandemics like COVID-19 [84,85,86,87].

## 8. Influence of the COVID-19 Pandemic on the Stress

The COVID-19 pandemic has had a significant impact on various aspects of social life and the mental health of people worldwide. A review of studies has shown that in the case of mothers of premature infants, COVID-19 has had a significant effect on exacerbating the already challenging situation of premature birth. This is a consequence of strong emotions and anxiety related to the risk of coronavirus infection for both the mother and, most importantly, the premature baby. Based on the conducted scientific research, it has been demonstrated that the COVID-19 pandemic has contributed to an increased level of anxiety and stress.

The main factors determining this phenomenon include:

- Physical and emotional stress: Mothers of premature infants experience both physical and emotional stress, resulting from the need to adapt to new requirements related to caring for a premature baby in conditions of an epidemiological threat. In a published Italian study from 2021 that focused on the impact of the COVID-19 pandemic on parental stress, breastfeeding, and lactation success among mothers of premature infants whose children were in the neonatal intensive care unit (NICU), it was found that the availability of breast milk and breastfeeding decreased drastically (prior to the pandemic, the percentage of premature infants breastfed with breast milk was 86.1%, while during the pandemic, it was 44.8%). However, it was noted that the rate of breastfeeding was similar before and during the pandemic after the premature baby’s discharge from the hospital. Another study conducted by the University of Liverpool and the School of Health in Australia examined experiences related to the stress of prematurity using the social media platform Twitter. A total of 3161 tweets from parents of premature infants were analyzed. It was found that the COVID-19 pandemic and the associated restrictions, which affected parents’ contact with preterm infants, imposed significant emotional burdens on the studied group [76,80,88,89,90,91,92,93,94,95].
- Health safety concerns: Mothers of premature infants have heightened concerns about their children’s health in the context of the pandemic. These concerns primarily revolve around the risk of coronavirus infection, especially in the case of premature babies who are more susceptible to infections. This leads to increased anxiety and fear in women who have given birth before the due date. Spanish scientists from Torrecardenas University Hospital (between 2020 and 2022) focused on assessing the kangaroo mother care method in reducing anxiety and stress in women after preterm birth in the context of the COVID-19 pandemic. A cohort study was conducted on recruited mothers whose children were in the neonatal intensive care unit and participated in kangaroo mother care, and their stress levels were measured using the Parental Stressor: Neonatal Intensive Care Unit (PSS: NICU) scale and the STAI E/R questionnaire before and after implementing kangaroo care. It was proven that mothers in the group where kangaroo care had not yet been applied obtained higher scores on the PSS: NICU scale and the STAI E/R questionnaire compared to women who practiced kangaroo care. However, no statistically significant differences were found (*p* > 0.05). The result of this study highlights the importance of kangaroo care for the mental health of mothers, which was often impossible or limited during the COVID-19 pandemic. According to WHO, Kangaroo mother care has been demonstrated to reduce baby mortality by as much as 40%, hypothermia by more than 70%, and severe infections by 65% in infants delivered preterm or at low birthweight [76,80,88,89,90,91,92,93,94,95].
- Restrictions on accessing medical care: The COVID-19 pandemic and the associated restrictions significantly hindered access to medical care and contact with doctors. The difficult access to specialized medical care for premature infants, such as specialist doctor visits, rehabilitation, or diagnostics, created a higher level of stress for parents of preterm newborns. At the beginning of the pandemic noticed that clinics and rehabilitation centers suspended their operations during the lockdown, follow-up appointments, therapy, and psychological support services have been discontinued in many locations around the world. This has many parents extremely concerned for the unpredictable effects on their child’s health [76,80,88,89,90,91,92,93,94].
- Separation of the premature baby from parents: Uncertainty related to coronavirus infection and its health consequences necessitated extraordinary precautions and safety measures in medical facilities. During the initial phase of the pandemic, mothers were completely isolated from their newborn babies and often saw them for the first time after completion of treatment in the NICU. This prolonged separation caused significant anxiety and stress in mothers of premature infants [76,80,89,90,91,92,93,94].
- Altered care conditions: Due to the pandemic, the standard procedures for care in neonatal intensive care units (NICUs) had to be modified to ensure the safety of both healthcare staff and patients. These changes, such as the use of personal protective equipment (PPE) and limitations on visitation, created additional challenges and emotional strain for parents, particularly mothers of premature infants [76,80,89,90,91,92,93,94].

## 9. Conclusions

**Figure 2:**
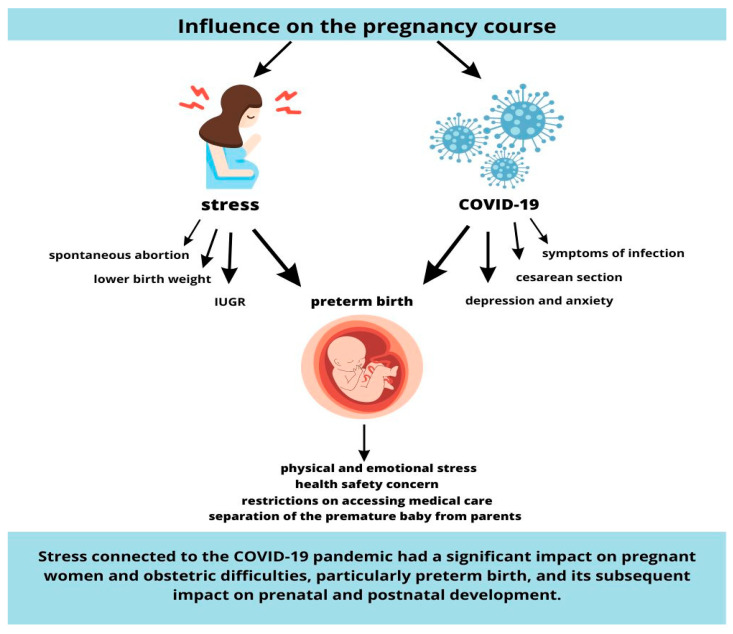
The interrelationships between stress, the COVID-19 pandemic and preterm birth.

In summary, the COVID-19 pandemic has had a profound impact on mothers of premature infants, exacerbating their experiences related to premature birth. The interrelationships between stress, the COVID-19 pandemic and prematurity are presented in Figure 2.

The physical, emotional, and practical challenges faced by these mothers have been further complicated by the pandemic, resulting in increased levels of stress and anxiety. Certainly there is a significant impact of limited access to health service, hospitalization and deferred diagnostic, treatment or rehabilitation of premature children on their future outcomes.

The limitation of this study is primarily the structure of the narrative review with a more descriptive and subjective view of the topic. Another limitation is the relatively short time that has elapsed since the beginning of the COVID-19 pandemic. For this reason, the long-term consequences for mothers and their offspring of premature delivery during the pandemic are currently unknown. However, an in-depth analysis of the literature allows us to summarize the current state of knowledge in many aspects of the subject. The article characterized the general impact of the COVID-19 pandemic on maternal and fetal health during pregnancy. In addition, stress is presented as a reaction at the physiological and molecular level, its impact on the prenatal and postnatal period of children. Furthermore, the study described a possible consequence of stress and the COVID-19 pandemic, caused by, among others, vitamin D deficiency, which is premature birth and the impact of prematurity on the future development of offspring.

More research is needed to investigate and observe how the stress of prematurity during the COVID-19 pandemic influences the long-term impact.

## Data Availability

Not applicable.
